# Relationship between Metabolic Syndrome and Its Components with Psychological Distress

**DOI:** 10.1155/2014/203463

**Published:** 2014-02-05

**Authors:** Hamidreza Roohafza, Masoumeh Sadeghi, Mina Naghnaeian, Pedram Shokouh, Abdollah Ahmadi, Nizal Sarrafzadegan

**Affiliations:** ^1^Isfahan Cardiovascular Research Center, Isfahan Cardiovascular Research Institute, Isfahan University of Medical Sciences, Isfahan 8187698191, Iran; ^2^Cardiac Rehabilitation Research Center, Isfahan Cardiovascular Research Institute, Isfahan University of Medical Sciences, P.O. Box 81465-1148, Isfahan 8187698191, Iran

## Abstract

*Background.* Metabolic syndrome (MetS) and psychological distress are hypothesized to have a bidirectional relationship. According to their high prevalence in most populations, appraisal of this theory would be of great clinical and research interest. *Methods.* Data were available as part of the Isfahan Healthy Heart Program (IHHP). A total of 9553 men and women aged ≥19 years from three counties in central Iran were selected. Measurements consisted of serologic tests, anthropometrics, and self-reported 12-item general health questionnaire. Logistic regression analysis was used to find the association between MetS, MetS components, and distress level. *Results.* The mean age of 9553 participants (50% male) was 38.7 ± 15.8 years. After adjusting for demographic factors, MetS (OR = 1.25, 95% CI: 1.01–1.37), central obesity (OR = 1.40, 95% CI: 1.15–1.49), and hypertension (OR = 1.55, 95% CI: 1.42–1.70) were associated with high distress level. However, after adding smoking status and low-density lipoprotein cholesterol to the adjustment factors, hypertension (OR = 1.79, 95% CI: 1.53–1.98) and central obesity (OR = 1.41, 95% CI: 1.17–1.55), but not the MetS, remained significantly associated with distress level. *Conclusion.* The presence of association between the MetS as well as its key components and high distress level signifies the importance of integrating psychological assessment and intervention in the standard management of MetS patients.

## 1. Introduction

Assuming that psychological distress affects all physiological systems in the body, researchers have focused on the role of psychological distress in the pathophysiology of noncommunicable diseases with almost unknown origin [1]. A number of psychological problems such as depression, anxiety, and psychological distress have been found to be related to chronic metabolic abnormalities, for example, insulin resistance, type-2 diabetes mellitus, and dyslipidemia [2]. Metabolic syndrome (MetS) is a combination of cardiovascular risk factors such as impaired glucose tolerance, dyslipidemia, hypertension, and central obesity that predisposes affected persons to cardiovascular morbidity and mortality [3]. A full image of the contributing factors to the syndrome has not yet been mapped [4].

Reports signifying the coincidence of a high prevalence of MetS [5–7] and psychological distress [8–10] in both developing and developed countries have raised the possibility of a relationship between distress and the metabolic derangements of the MetS. In the same context, prior studies have provided evidence for assuming prolonged exposure to chronic psychological distress (in form of daily life stressors) as an important risk factor for MetS [11]. However, when taken altogether, the published data are scant and mixed. Some authors reported positive associations [12, 13], while others obtained contradicting results [14]. It seems that associations with distress level are more likely to be found when considering the components rather than the construct of the MetS.

The present study employed a representative community-based sample to unveil associations between the whole MetS as well as its individual components with the level of psychological distress.

## 2. Materials and Methods

### 2.1. Study Population

Subjects' data were available as part of the Isfahan Healthy Heart Program (IHHP), which is a community-based program designed to prevent and control cardiovascular diseases. The IHHP promotes healthy nutrition and physical activity and conducts tobacco control and distress management programs. Further details of this study are described elsewhere [15].

A total of 9553 men and women aged ≥19 years from three counties in central Iran (Isfahan, Najafabad, and Arak) were selected according to the national census in 2007. In each urban or rural community, a random sample of adults was selected by multistage cluster sampling. Approximately, 5–10% of households within these clusters were randomly selected for inclusion. The number of participants for this study was determined according to their sex and age compared with the entire population. To achieve an adequate sample size, those who declined to participate in the study were replaced by their neighbors. Pregnant and mentally retarded or physically disabled individuals were not eligible for enrollment.

Individuals underwent a 45-minute home interview carried out by a trained health professional. The interview included information on demographics, smoking status, and psychological distress. A written informed consent was also obtained from all individuals prior to participating in the study. The research protocol was approved by the ethics committee of the Isfahan University of Medical Sciences.

### 2.2. Demographic and Serologic Assessments

The recorded demographic characteristics consisted of age, gender, educational level (classified as 0–5 years, 6–12 years, or >12 years), and marital status (married versus unmarried including single, widowed, or divorced). Current smokers were defined as individuals who reported to smoke at least one cigarette per day. MetS was diagnosed according to the National Cholesterol Education Program Adult Treatment Panel III (ATP III) criteria [16].

All individuals were invited to the nearest health center to their places of residence for doing blood sampling and measurements. A venous blood sample was taken after at least 12 hours of fasting through the antecubital vein to measure fasting plasma glucose (FPG), triglycerides (TG), low-density lipoprotein cholesterol (LDL-C), and high-density lipoprotein cholesterol (HDL-C) levels. TG, FPG, and HDL-C were quantified by an enzymatic method using Elan 2000 autoanalyzer. Friedewald formula was used for calculating LDL-C level except in individuals with TG ≥ 400 mg/dL where LDL-C level was measured directly. All assays were performed in the central laboratory of the Isfahan Cardiovascular Research Centre. Before blood sampling, arterial blood pressure measurement was done for all participants in the sitting position twice with an interval of 15 minutes and finally the mean of 2 records was used for analysis. Waist circumference (WC) was gauged by tape measure horizontally 1 cm above the navel.

### 2.3. Psychological Distress

Psychological distress was measured by a 12-item General Health Questionnaire (GHQ-12), a well-established screening tool in assessing psychological distress [17]. There is evidence that the GHQ-12 is a consistent and reliable instrument for using in general population studies [18]. Each item is rated on a four-point scale (less than usual, no more than usual, fairly more than usual, or much more than usual). The system used to score the GHQ-12 questionnaires in this study was the GHQ score method (0-0-1-1 method). Using this method, a participant could score between 0 and 12, and a threshold score of equal to or more than of four has been used to identify high distress level individuals.

### 2.4. Statistical Analysis

Data entry was done using Epi Info, Version 6 (Centers for Disease Control, Atlanta, GA). All data were analyzed using the SPSS version 15 (SPSS Inc, Chicago, IL). A *P* value of ≤0.05 was considered statistically significant in all analyses. Student's *t*-test for continuous and Chi-square test for discrete variables were used.

Logistic regression analysis was performed to study the association between the MetS and its components and distress level. Independent variables included MetS and its five components as well as smoking status, LDL-C, age, sex, and educational level, and dependent variable was distress level. Odds ratios (ORs) were reported with the corresponding 95% confidence intervals (CIs). ORs were presented as “unadjusted,” “model 1” (adjusted for age, sex, and educational level), and “model 2” (adjusted for age, sex, educational level, smoking status, and LDL-C).

## 3. Results

Demographic characteristics analysis demonstrated that individuals with high distress level were significantly older (*P* = 0.001) and more frequently women (*P* ≤ 0.001) (Table 1). On the other side, individuals in low distress group were more likely single and educated than high distress group (*P* ≤ 0.001). Significantly, high distress subjects were more probable to smoke than others (*P* = 0.01). As shown in Table 1, MetS tended to affect high distress group more (*P* = 0.003). Nevertheless, high LDL-C levels (defined as ≥130 mg/dL) did not seem to be influenced by the level of distress.

Respectively, in high distress group, the prevalence of having 0, 1, 2, 3, 4, and 5 MetS components was 23.6%, 30.3%, 26.3%, 15%, 5.8%, and 1.5%, which were considerably different than 22.4%, 29.8%, 25.6%, 13.4%, 5.5%, and 0.9% in low distress group (*P* = 0.022).

With respect to the association between distress status and the presence of MetS, individuals identified as having MetS are at an increased risk of having higher levels of distress (OR = 1.15 95% CI; 1.04–1.28); this risk also remained significant after adjusting for age, sex, and educational level (OR = 1.25 95% CI; 1.01–1.37). Notably, the association lost significance after more adjusting for smoking status and LDL-C (OR = 1.31 95% CI; 1–1.49).

The presence of hypertension was significantly associated with high distress status in both model 1 (OR = 1.55, 95% CI; 1.42–1.70) and model 2 (OR = 1.79; 95% CI = 1.53–1.98). Having central obesity was also significantly related to high distress status even after adjusting for age, sex, educational level, smoking status, and LDL-C (OR = 1.41 95% CI; 1.17–1.55). Although HDL cholesterol was significantly associated with high distress status before adjusting (OR = 1.14 95% CI; 1.04–1.24), no significant relation was found in both model 1 and model 2. However, Fasting plasma glucose and triglycerides were not significantly associated with distress level.

## 4. Discussion

This study showed that MetS and two of its components, central obesity and hypertension, are significantly associated with high distress status even after adjustment for other covariates.

At present, there is an increasing interest in the relationship between MetS and distress and whether or not a causal relationship exists. Previous studies have shown an elevated prevalence of MetS among patients having high distress levels and especially those with major depression [9]. It was concluded that distress at work is an important risk factor for the MetS [19]. A research in Finland also provided further support for the theory connecting psychological and metabolic abnormalities [20]. Trying to explain this association pathophysiologically, Björntorp and Rosmond have hypothesized that stressful events are linked to metabolic disorders via visceral fat accumulation [21]. It has been suggested that the MetS might be induced through the elevated cortisol secretion level which occurs during reactions to perceived distress in everyday life. Naturally, phenotypic variations are expected as a result of gene-environment interactions [21]. On the other hand, our findings were in favor of the existence of no detectable link between having ≤2 components of MetS and high distress status, which are consistent with the data obtained from a Taiwanese population [22].

According to what we observed, abdominal obesity, a key component of the MetS, is affected by distress. Additional evidence from longitudinal studies suggests that chronic life stress may be causally linked to weight gain [23]. Conversely, it cannot be excluded that being overweight contributes to psychological distress [24, 25]. This argument would practically put forward a bidirectional relationship. Contradicting the larger body of positive evidence [12, 13], few reports are available claiming no association [14]. Reviewers have speculated that these inconsistent results are possibly indicative of specific subgroups among the obese population [26].

It has been suggested that chronic distress exposure, such as long-term work in a high job strain environment or in a context with low social support, is related to increased 24-hour ambulatory blood pressure and cardiac morbidity [27]. The considerable odds ratio of high level of distress for having hypertension in our study also provided further support for this theory. However, results of a few studies linking hypertension to psychological status remain at issue due to being biased by such factors as awareness of hypertension and/or imperfections in measuring blood pressure [28].

It is not unreasonable to deem oxidative stress as one of the underlying mechanisms relating high distress status to the MetS and coronary artery disease. Being implicated in the pathogenesis of diverse disease states, oxidative stress was blamed to be a shared pathology between high distress status and some major psychiatric disorders such as depression [29, 30]. Recently, there have been attempts to define the contribution of individual components of the MetS to oxidative stress in MetS patients. Obesity, as a core component, plays a central role in the amplification of oxidative stress. The same is true for the hypertension, which is independently associated with increased levels of oxidative stress [31]. Nevertheless, a research by Tsuboi et al. showed the other MetS components (low HDL-C and high triglycerides and FPG) to have minimal contributions [30].

There is at least some evidence suggestive for the mediating role of low-grade inflammation in the link between distress and MetS. Inflammation was found to be independently associated with psychological distress even when controlled for the presence of the MetS [32]. Furthermore, it is well documented that low-grade inflammatory state has a strong relationship with enlarged adipose tissue. An association that stems from the chronic activation of innate immune system in the white adipose tissue [33]. Notably, inflammatory processes are also shown to be present in the vasculature of hypertensive subjects, pointing to their role in the pathophysiology of hypertension [34].

Although benefited from a sample wide in age range and sufficient in size, the present study is hampered by a number of limitations. First is the possibility of recall bias, which might occur because participants with MetS may report poorer social and emotional situation. In addition, cross-sectional design of the study implies limited power to explore cause-and-effect relationships.

## 5. Conclusion

In summary, we indicated that other than two important components of MetS, abdominal obesity and hypertension, the construct of the syndrome is associated with high distress status. Thus, it seems feasible to monitor and manage the presence of MetS in high distress individuals, which enables an early intervention, and ultimately, attenuates the development of burdensome complications of the MetS including cardiovascular disease.

## Figures and Tables

**Table 1:** Baseline characteristics according to distress level.

	High distress (*n* = 3264) *N* (%)	Low distress (*n* = 6289) *N* (%)	*P* value
Demographics			
Age (mean ± SD)	39.51 ± 16.24	38.31 ± 15.11	0.001
Sex			
Male	1375 (42.1%)	3402 (54.1%)	<0.001
Female	1889 (57.9%)	2887 (45.9%)	
Educational level			
0–5 y	1622 (49.7%)	2680 (42.6%)	<0.001
6–12 y	1279 (39.2%)	2704 (43.0%)	
>12 y	363 (11.1%)	905 (14.4%)	
Marital status			
Married	2442 (74.8%)	4981 (79.2%)	<0.001
Single	822 (25.2%)	1308 (20.8%)	
Current smoking	474 (14.5%)	811 (12.9)	0.015
LDL cholesterol ≥ 130 mg/dL	1114 (34.1%)	2057 (33.2%)	0.173
Metabolic syndrome	719 (22.0%)	1232 (19.8%)	0.003

Metabolic syndrome components			
Fasting plasma glucose ≥ 100 mg/dL	258 (7.9%)	458 (7.3%)	0.281
Triglycerides ≥ 150 mg/dL	1128 (34.6%)	2278 (36.2%)	0.051
HDL cholesterol (male < 40 mg/dL, female < 50 mg/dL)	1807 (55.4%)	3285 (52.2%)	0.002
Waist circumference (male > 102 cm, female > 88 cm)	1020 (31.2%)	1795 (28.5%)	<0.001
Blood pressure ≥ 130/85 mm Hg	852 (26.1%)	1519 (24.1%)	0.019
